# Management Dilemma: Myeloperoxidase (MPO)-Positive Vasculitis in a Patient With Metastatic Malignant Melanoma

**DOI:** 10.7759/cureus.105864

**Published:** 2026-03-25

**Authors:** Aarush Sajjad, Ashwin Manivannan

**Affiliations:** 1 Internal Medicine, Whiston Hospital, Rainhill, GBR; 2 Cardiology, Whiston Hospital, Rainhill, GBR

**Keywords:** anca-associated vasculitis, cancer immunotherapy, eosinophilic granulomatosis with polyangiitis, immune-checkpoint inhibitors, metastatic malignant melanoma, mpo-positive vasculitis, myeloperoxidase-positive vasculitis, vasculitic mononeuropathy

## Abstract

A man in his 80s with a history of eosinophilic granulomatosis with polyangiitis (EGPA) presented with progressive bilateral leg weakness, sensory loss, and pain, on a background of metastatic malignant melanoma awaiting pembrolizumab therapy. Investigations revealed a relapse of anti-neutrophil cytoplasmic antibody (ANCA)-associated vasculitis, manifesting as vasculitic mononeuropathy. This created a therapeutic dilemma: corticosteroids were required to control vasculitis but risked blunting the effect of imminent immune checkpoint inhibition, highlighting the need for multidisciplinary management.

The patient was diagnosed with EGPA in 2008 following presentation with adult-onset asthma, peripheral eosinophilia, and neuropathic symptoms. He achieved sustained remission after induction therapy with intravenous cyclophosphamide followed by a prolonged tapering course of oral corticosteroids and had remained clinically quiescent for over a decade.

In 2023, he was diagnosed with metastatic malignant melanoma with liver and suspected pulmonary metastases and was referred for immune checkpoint inhibitor therapy (pembrolizumab), which had not yet been initiated at the time of this admission. Approximately 15 years after his initial EGPA diagnosis - and prior to receiving any immunotherapy - he developed progressive lower limb weakness and sensory symptoms, prompting the current admission and revealing a relapse of myeloperoxidase (MPO)-positive ANCA-associated vasculitis.

## Introduction

Anti-neutrophil cytoplasmic antibody (ANCA)-associated vasculitis (AAV) is a group of small-vessel autoimmune disorders characterised by antibodies to myeloperoxidase (MPO) or proteinase-3 (PR3). Eosinophilic granulomatosis with polyangiitis (EGPA) represents the least common subtype, with an incidence of 1-3 per million annually and significant morbidity when uncontrolled. However, the incidence rate of AAV in general appears to have increased over the years, possibly due to the improvements in ANCA testing and recognition of clinical presentations [1]. For EGPA, around 30% of cases are anti-MPO-positive, 10% of cases are anti-PR3, and 60% are ANCA-negative. Checkpoint inhibitors such as pembrolizumab activate antitumour immunity but can also exacerbate or induce autoimmune disease, including vasculitis. Managing both conditions simultaneously presents a therapeutic paradox: immunotherapy can trigger vasculitis flares, while steroids and other immunosuppressants can diminish tumour response [2]. This case illustrates how such competing imperatives were balanced in practice. 

The 2022 American College of Rheumatology/European Alliance of Associations for Rheumatology (ACR/EULAR) classification criteria for EGPA incorporate weighted clinical, laboratory, and histological features, including asthma, nasal polyps, peripheral eosinophilia, neuropathy, pulmonary infiltrates, and biopsy-proven vasculitis, generating a score ≥six to classify EGPA with high specificity. ANCA positivity is not required, but identifies a distinct clinical subset characterised by more frequent vasculitic manifestations such as glomerulonephritis and mononeuritis multiplex [3]. This case adds to the literature by describing relapse of MPO-positive vasculitis occurring prior to immune checkpoint inhibitor (ICI) initiation in a patient with metastatic melanoma, highlighting the need for baseline autoimmune vigilance and multidisciplinary planning.

## Case presentation

A man in his 80s presented with a two-week history of worsening bilateral leg weakness, numbness, and hip pain radiating down both legs. There was no trauma, sphincter disturbance, or saddle anaesthesia. Past medical history included EGPA diagnosed in 2008 (treated with intravenous cyclophosphamide), late-onset asthma, and metastatic malignant melanoma (staging pT4b) with liver and possible lung metastases, awaiting immunotherapy. He had been treated in the community for presumed polymyalgia rheumatica with prednisolone 20 mg daily, but had not been able to taper below this dose due to ongoing symptoms.

**Figure 1:**
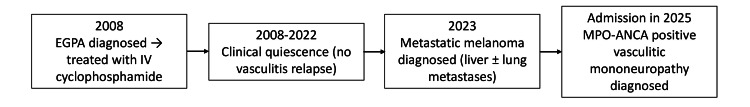
Timeline of EGPA disease course, melanoma diagnosis, and subsequent vasculitic relapse EGPA: eosinophilic granulomatosis with polyangiitis; MPO: myeloperoxidase; ANCA: anti-neutrophilic cytoplasmic antibody; IV: intravenous. Image credit: Created by the authors using Microsoft PowerPoint (Microsoft Corp., Redmond, WA, USA).

On examination, tone & reflexes were preserved, but power was markedly reduced (R upper limb 2/5, L upper limb 4/5; R lower limb 3/5, L lower limb 4/5). There was no rash, haemoptysis, renal or ear, nose, and throat (ENT) involvement. There was no history of haemoptysis, epistaxis, sinus symptoms, hearing loss, haematuria, or skin rash, and no clinical features suggestive of renal, ENT, or pulmonary vasculitic involvement. The timeline of the disease course is given in Figure 1. 

Investigations

Key initial investigations are summarised in Table 1 below:

**Table 1:** Key investigations

Test	Result	Reference range
WCC (White Cell Count)	22.2 × 10^9^/L (neutrophils 20.3, eosinophils 0.0)	4–11 × 10^9^/L (neutrophils 2-7.5× 10^9^/L, eosinophils 0-0.4x 10^9^/L)
CRP (C-Reactive Protein)	13 mg/L	<5 mg/L
ESR (Erythrocyte Sedimentation Rate)	45 mm/h	<20 mm/h
p-ANCA (perinuclear-Anti-Neutrophilic Cytoplasmic Antibody)	Positive	Negative
MPO (Myeloperoxidase) antibody	>134 IU/mL	<6 IU/mL
Protein:creatinine ratio	159 mg/mmol	<50 mg/mmol
Urea	6.9mmol/L	2.5-7.8mmol/L
Creatinine	64umol/L	65-104umol/L

Due to the elevated MPO, an indirect immunofluorescence (IIF) assay was performed to investigate for ANCA - which came back positive for perinuclear-ANCA (pANCA). The remainder of the vasculitis screen was unremarkable, aside from a mildly decreased IgM level. No urine dip performed.

**Figure 2:**
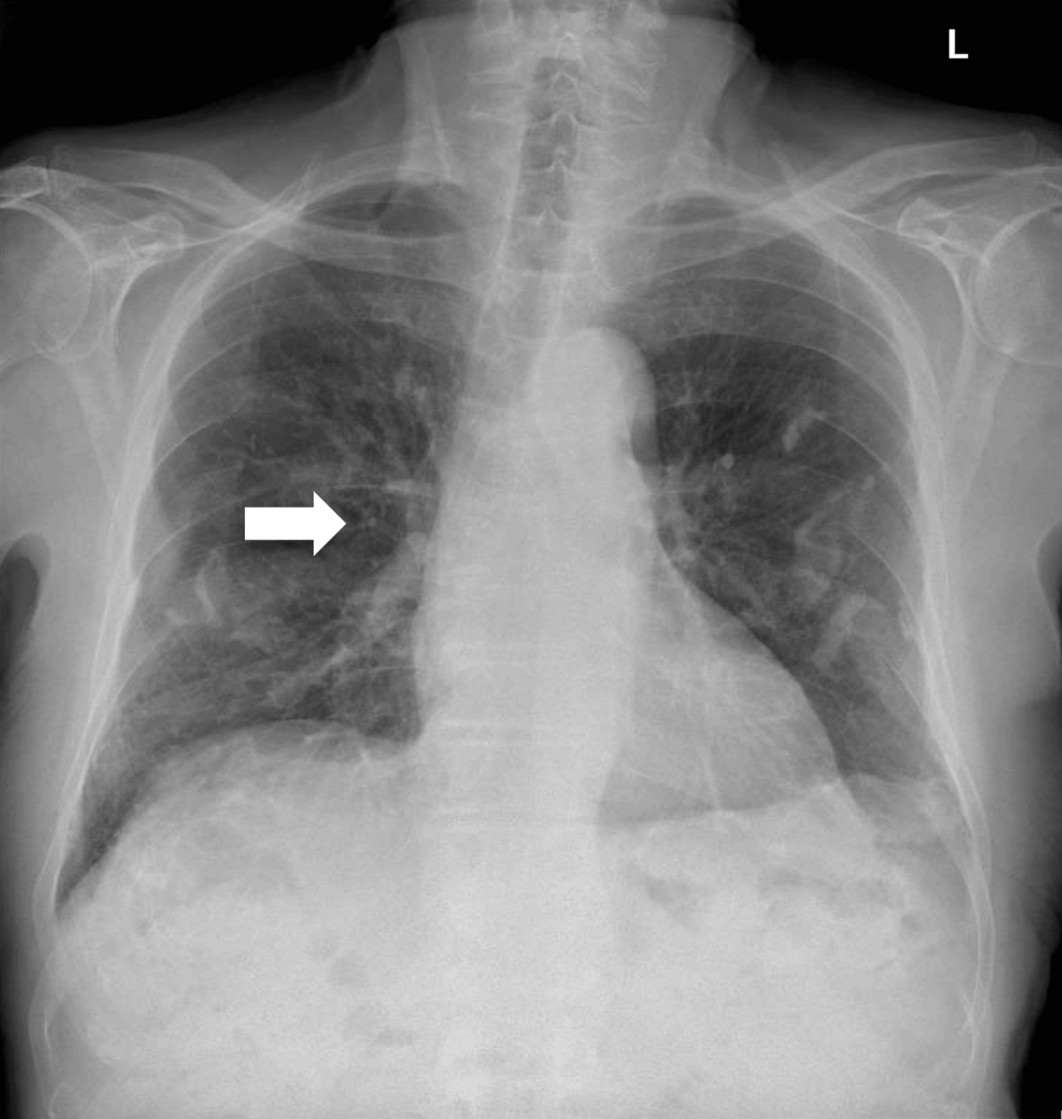
Chest X-ray on admission White arrows highlight longstanding bilateral pleural plaques. The arrowhead indicates a 6 mm right hilar nodule consistent with known metastatic disease.

A chest X-ray was performed as part of a septic screen to rule out an infection as a cause of his raised inflammatory markers, as well as ruling out pulmonary haemorrhage due to his relevant medical history (Figure 2).

**Figure 3:**
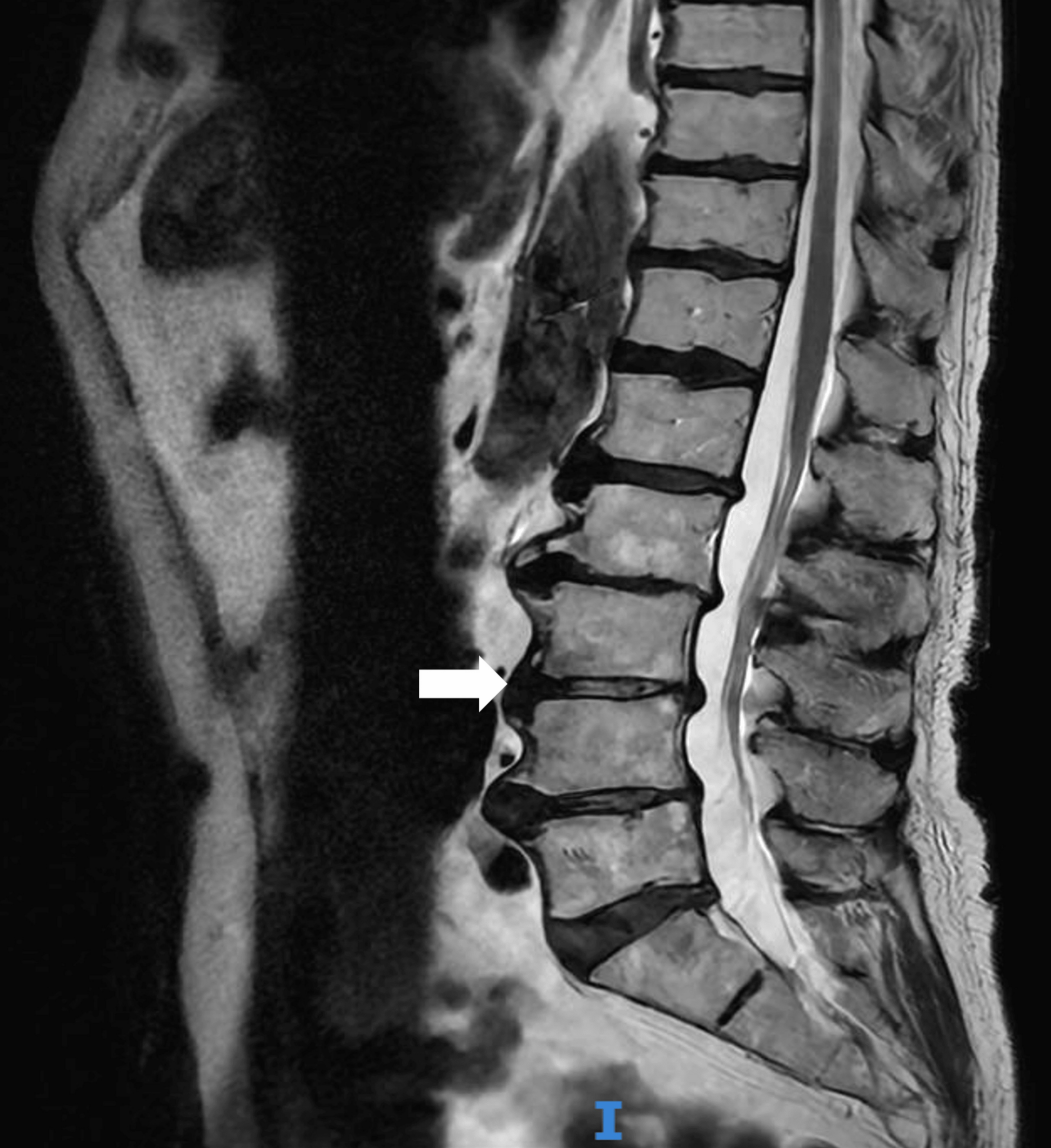
Sagittal T2-weighted MRI lumbar sacral spine Shows multilevel disc bulge extending from L1 to S1 with multiple impinged nerves, most significant at L3/L4.

Due to his presentation of neurological symptoms and background of a metastatic malignancy, an MRI of the lumbosacral spine was performed to investigate for metastatic cord compression (MSCC) (Figure 3).

This revealed multilevel disc bulges, but no metastatic cord compression. Nerve-conduction studies confirmed vasculitic mononeuropathy of the peroneal nerve, specifically demonstrating severe generalised sensory/motor large fibre peripheral neuropathy of the axonal type. ⁠There was marked active denervation changes noted in bilateral tibialis anterior muscles (right more than left), raising the possibility of vasculitic mononeuropathy involving the peroneal nerves.

His inflammatory markers rose transiently during admission, with C-reactive protein peaking at 118 mg/L (reference value: <5 mg/L), but no infective source was identified. Peripheral blood cultures showed no growth in aerobic or anaerobic bottles, and there were no clinical features of sepsis. The persistent neutrophilia was therefore felt most likely to be secondary to corticosteroid therapy rather than infection. Interestingly, although a diagnosis of EGPA is commonly associated with eosinophilia, the patient's eosinophils were not raised most likely due to long-term steroid use, which tends to suppress the production of inflammatory mediators and recruitment of eosinophil.

## Discussion

Differential diagnosis

Initial diagnostic considerations included relapse of EGPA or other ANCA-associated vasculitis, paraneoplastic neuropathy related to metastatic melanoma, and neurological symptoms secondary to metastatic disease or spinal cord compression. Formal disease activity scoring (e.g., Birmingham Vasculitis Activity Score (BVAS)) was not prospectively calculated; however, the patient demonstrated clinically active disease based on new peripheral neuropathy. Metastatic cord compression was excluded on MRI, and the pattern of neuropathy, together with strongly positive MPO antibodies and p-ANCA on immunofluorescence, supported a diagnosis of vasculitic mononeuropathy rather than paraneoplastic disease [3]. The patient had obstructive airway disease, evidence of motor neuropathy not due to radiculopathy, and nasal polyps - all of which are criteria in the 2022 ACR/EULAR scoring system.

Treatment

Rheumatology were consulted, who advised treatment with IV methylprednisolone with oral prednisolone tapering, instead of rituximab. Intravenous (IV) methylprednisolone 500 mg was commenced for three days and followed by a reduced-dose glucocorticoid taper broadly aligned with the Plasma EXchange and glucocorticoids In anti-neutrophil cytoplasm antibody associated systemic VASculitis (PEXIVAS) regimen. This consisted of 60 mg prednisolone PO initially, and to taper by 5 mg weekly to his maintenance dose of 20 mg OD. Although PEXIVAS did not specifically study EGPA, this approach was chosen to minimise cumulative steroid exposure in an elderly patient with active malignancy while maintaining adequate disease control for organ-threatening vasculitic neuropathy. Bone and gastric protection were also initiated. Immunotherapy was deferred pending the multidisciplinary discussion between oncology and rheumatology teams because of the risk that pembrolizumab might exacerbate vasculitis. Positron Emission Tomography - Computed Tomography (PET-CT) for malignancy staging was postponed, as the steroid dosing could easily mask the possible vasculitis changes on the scan [4].

Outcome and follow-up

Neurological power and sensation improved following steroid therapy, with restoration of full strength in all four limbs prior to discharge. The patient was referred to the regional rheumatology-oncology multidisciplinary team for ongoing management planning. As he was relocating to another region of the country, care was formally handed over to the relevant rheumatology and oncology services at the receiving Trust to ensure continuity of immunological and oncological follow-up, including decisions regarding initiation of ICI therapy. Also, given stable renal function throughout admission, there was no consideration of renal biopsy.

Literature review

This literature review was conducted as a targeted narrative review rather than a formal systematic review. PubMed was searched for keywords (“EGPA”, “ANCA-associated vasculitis”, “immune checkpoint inhibitor”), and relevant peer-reviewed articles were selected to contextualise the clinical scenario.

High-dose corticosteroids are central to vasculitis control but may reduce the effectiveness of ICI by dampening T-cell activation. Conversely, immunotherapy can precipitate severe autoimmune flares. Published evidence suggests that, in patients with pre-existing autoimmune disease, ICI therapy can be feasible provided the underlying condition is controlled and patients are closely monitored. Small-vessel vasculitis has been reported both as a de novo immune-related adverse event and as a flare of established disease following Programmed cell death protein 1 (PD-1) blockade [5-7].

ICIs, particularly PD-1 inhibitors, have been increasingly associated with de novo vasculitis and flares of pre-existing autoimmune disease, including small-vessel vasculitis and mononeuritis multiplex [8]. ANCA-associated vasculitis has been reported following ICI exposure, with manifestations including peripheral neuropathy and renal involvement [9].

Immune-related adverse events associated with checkpoint inhibitors can extend beyond classical organ-specific toxicity and include systemic autoimmune phenomena, complicating diagnostic assessment and therapeutic sequencing [10]. The clinical phenotypes of AAV consist of EGPA, granulomatosis with polyangiitis (GPA), microscopic polyangiitis (MPA) and renal-limited vasculitis [11].

More recently, the British Society for Rheumatology (BSR) published updated recommendations for management of AAV in 2025, including EGPA, to induce and maintain remission. Their management algorithm states that for induction of remission for life- or organ-threatening disease, cyclophosphamide or rituximab can be used in combination with glucocorticoids [12]. Rituximab may be favoured over cyclophosphamide in patients with childbearing potential. In non-severe EGPA, it is recommended to use more conventional immunosuppressive agents such as methotrexate, azathioprine or mycophenolate mofetil, alongside glucocorticoids [13]. 

Interleukin-5 (IL-5) monoclonal antibody therapy has been evaluated in EGPA. In a randomised trial, mepolizumab improved remission duration and reduced glucocorticoid use compared to standard care [13]. This was followed by another randomised controlled trial comparing the efficacy and safety of another IL-5 monoclonal antibody, benralizumab, with mepolizumab. This was carried out on 140 patients, with half receiving 30 mg benralizumab and the other half receiving 300 mg mepolizumab every four weeks for 52 weeks. There was no significant difference between both therapies in induction of remission, however both showed potential to reduce dependency on oral glucocorticoids [14]. As such, BSR recommends the use of anti-IL5 therapy, where available, for induction of remission in those with non-life or organ-threatening EGPA. With regards to maintaining remission, additional immunosuppression with a tapering dose of glucocorticoids has been recommended by BSR [12]. 

Besides the conventional immunosuppressive treatment algorithms that have been recommended, there has also been research into a newer agent, avacopan. It works by targeting the complement system and inhibiting C5a receptors, which are thought to be involved in the pathophysiology of AAV. A meta-analysis of nine studies evaluated the use of avacopan in the treatment for patients with AAV, being studied as both a monotherapy and in combination with rituximab or cyclophosphamide [15]. It demonstrated superior efficacy in sustaining remission and highlighted its potential as a steroid-sparing agent in reducing the burden of glucocorticoid-associated toxicity. However, this review does not distinguish its use in EGPA, GPA and MPA. Currently BSR only recommends its use in GPA and MPA [12].

Learning outcomes

Management requires careful balance in the setting of dual pathology. Active vasculitis should be adequately controlled before initiating ICI therapy. Steroid stewardship is critical, as high-dose corticosteroids may attenuate ICI efficacy; therefore, judicious tapering under multidisciplinary team oversight is essential. Close collaboration between rheumatology, oncology, and neurology optimises both autoimmune control and oncological outcomes. Ongoing clinical vigilance is required, as new neurological symptoms in patients with cancer may reflect vasculitic neuropathy rather than metastatic disease.

## Conclusions

This case illustrates the clinical tension between treating active vasculitis and preserving oncological efficacy in the era of ICIs. Our management strategy - stabilising vasculitis first, tapering steroids, and involving a joint multidisciplinary therapy - aligns with current pragmatic recommendations. This case underscores the need for formal protocols outlining baseline autoimmune assessment, thresholds for ICI initiation, and structured tapering plans. This case adds to the growing literature on autoimmune disease management in the immunotherapy era and underscores the need for prospective guidance on sequencing immunosuppression and immune checkpoint inhibition. Furthermore, this case emphasises that neurological deterioration in patients with malignancy should not be reflexively attributed to metastatic disease. In patients with a history of autoimmune vasculitis, relapse remains an important and treatable differential diagnosis, even years after apparent remission.

One aspect that is worth highlighting in this case is the deviation of the treatment from the recommended guidelines. The management of this patient’s vasculitis had added complexity due to the metastatic malignancy that was also under review for treatment. As such, having a multidisciplinary approach is of the utmost importance to ensure patient safety, and the decision of the specialist teams to opt for steroid-only management initially may have been in consideration of the pending immunotherapy. One significant limitation of this case report is that, unfortunately, with the patient relocating, we were not able to follow through on how his remission was maintained and what decision was made with regard to his immunotherapy. 
